# Congenital nephrogenic diabetes insipidus accompanied with central nephrogenic diabetes secondary to pituitary surgery -a case report

**DOI:** 10.1186/s12902-021-00749-y

**Published:** 2021-04-21

**Authors:** Wei Zhang, Yimin Shen, Yuezhong Ren, Yvbo Xin, Lijun Wang

**Affiliations:** 1 Department of Endocrinology, Zhejiang Provincial People’s Hospital, Affiliated People’s Hospital, Hangzhou Medical College, Hangzhou, 310003 Zhejiang province China; 2 Key Laboratory of Endocrine Gland Diseases of Zhejiang Province, Affiliated People’s Hospital, Hangzhou Medical College, Hangzhou, 310003 Zhejiang province China; 3 Department of Cardiology, The Second Affiliated Hospital of Zhejiang University School of Medicine, Hangzhou, 310009 Zhejiang province China; 4 Department of Endocrinology, The Second Affiliated Hospital of Zhejiang University School of Medicine, Hangzhou, 310009 Zhejiang province China

**Keywords:** Polyuria, Nephrogenic diabetes insipidus, Central nephrogenic diabetes, AVPR2 mutation

## Abstract

**Background:**

Diabetes insipidus (DI) can be a common cause of polydipsia and polyuria. Here, we present a case of congenital nephrogenic diabetes insipidus (CNDI) accompanied with central diabetes insipidus (CDI) secondary to pituitary surgery.

**Case presentation:**

A 24-year-old Chinese woman came to our hospital with the complaints of polydipsia and polyuria for 6 months. Six months ago, she was detected with pituitary apoplexy, and thereby getting pituitary surgery. However, the water deprivation test demonstrated no significant changes in urine volume and urine gravity in response to fluid depression or AVP administration. In addition, the genetic results confirmed a heterozygous mutation in arginine vasopressin receptor type 2 (AVPR2) genes.

**Conclusions:**

She was considered with CNDI as well as acquired CDI secondary to pituitary surgery. She was given with hydrochlorothiazide (HCTZ) 25 mg twice a day as well as desmopressin (DDAVP, Minirin) 0.1 mg three times a day. There is no recurrence of polyuria or polydipsia observed for more than 6 months. It can be hard to consider AVPR2 mutation in female carriers, especially in those with subtle clinical presentation. Hence, direct detection of DNA sequencing with AVPR2 is a convenient and accurate method in CNDI diagnosis.

## Background

Diabetes insipidus (DI) can be a common cause of polyuria [1]. Here, we describe a rare case of congenital nephrogenic diabetes insipidus (CNDI) observed after pituitary apoplexy. A 24-year-old Chinese woman came to our hospital with complaints of polydipsia and polyuria for 6 months. She was diagnosed with pituitary apoplexy 6 months ago, and pituitary surgery was performed. Later, she developed polyuria as well as polydipsia with a plasma osmolality of 301.00 mOsm/(kg·H2O) and urine gravity of 1.002. However, the water deprivation test demonstrated no significant changes in urine volume and urine gravity in response to fluid depression or AVP administration. The heterozygous mutation in arginine vasopressin receptor type 2 (AVPR2) genes further confirmed her as having CNDI. Therefore, she was given 25 mg hydrochlorothiazide (HCTZ) twice a day, 0.1 mg desmopressin (DDAVP, Minirin) three times a day and 10 mg methimazole twice a day. After that, no polyuria or polydipsia was detected. In addition, differential diagnoses of DI as well as the specific implications of AVPR2 mutations in nephrogenic diabetes insipidus (NDI) are discussed.

## Case presentation

**Fig. 1:**
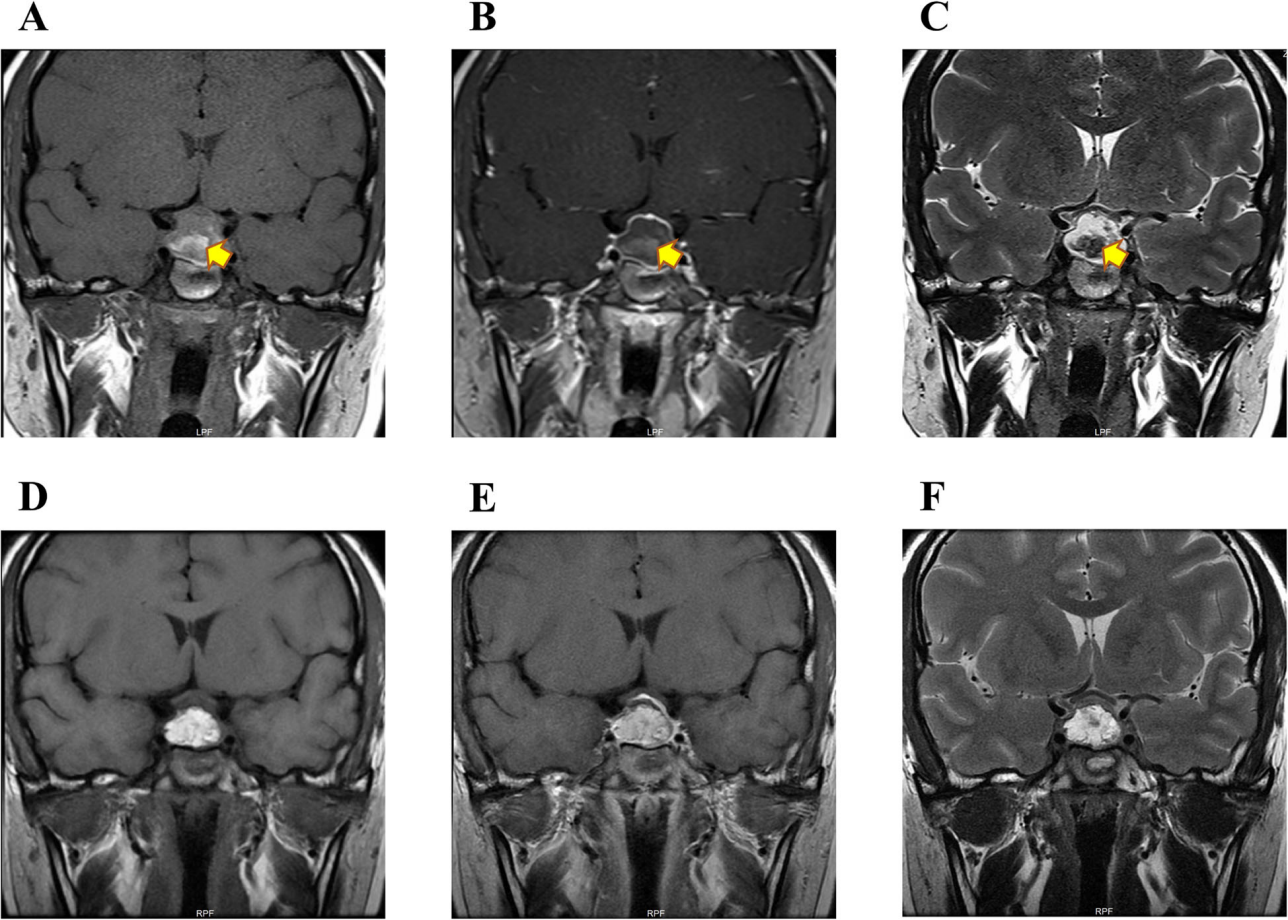
Coronal magnetic resonance imaging of pituitary in this patient. Before the surgery: pituitary apoplexy, with weakened signal in the posterior pituitary. A. T1; B. T1 + strengthen; C. T2. After the surgery: with stuffing added. D. T1; E. T1 + strengthen; F. T2

**Fig. 2:**
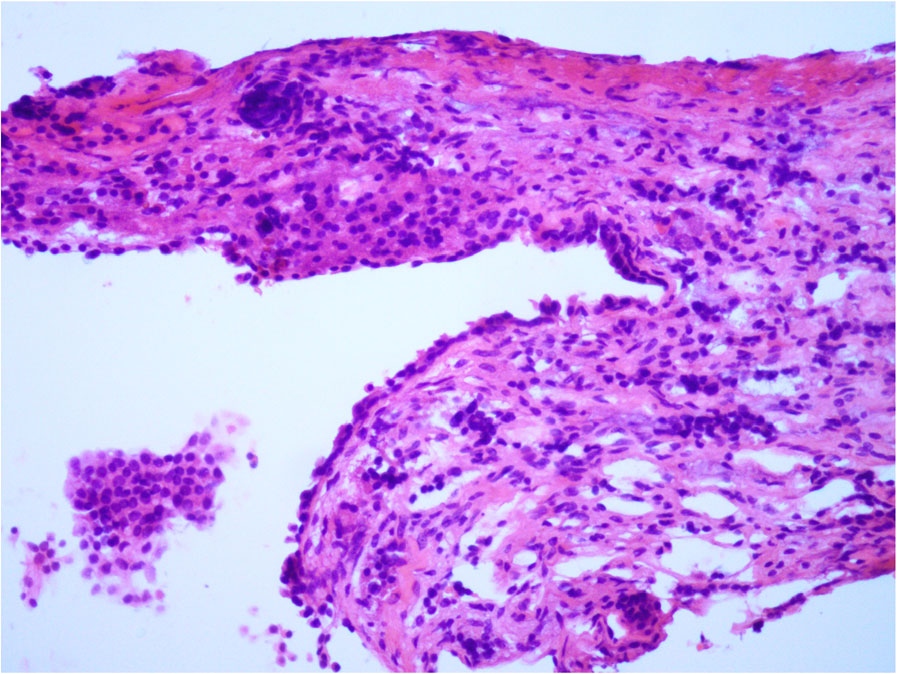
The pathological results of the resected pituitary mass. It was suggested with nodular hyperplasia with hemorrhage and exudation. There was no structural necrosis and a few lymphocytes infiltrated in powder staining. Several areas presented with adenomatoid changes, which were consistent with Rathke cyst

In January 2020, a 24-year-old Chinese woman presented to the Department of Endocrinology with complaints of persistent polydipsia as well as polyuria for nearly 6 months. Six months ago, she was admitted to the neurosurgery department because of headache, and magnetic resonance imaging (MRI) determined pituitary apoplexy, with weakened signal in the posterior pituitary **(**Fig. 1**)**. Later, she was treated with transnasal transsphenoidal resection for the pituitary mass, and the pathology findings suggested pituitary adenoma **(**Fig. 2**).** She was then prescribed with methylprednisolone 40 mg once after pituitary surgery, and her cortisol level (8 am-4 pm-12 pm): 143.13μg/L-47.12μg/L-5.93μg/L, adrenocorticotropic hormone (ACTH) level (8 am-4 pm-12 pm): 33 pg/mL-18.8 pg/mL- < 5 pg/mL, 24-h urine cortisol level was 388μg, as well as gonadotrophins and thyroid hormones were all in the normal range at that period. Later, she developed severe polydipsia as well as polyuria, generally urinating every 2 h with 10 L per day in total. There was no backache, hand tremors, blurred vision, lower limb puffiness or weight loss. The laboratory examination revealed that the plasma osmolality was 301.00 mOsm/L, urine gravity was 1.002, potassium was 4.17 mmol/L, and serum sodium was 138.5 mmol/L. Then, she was diagnosed with pituitary surgery-derived central diabetes insipidus (CDI). However, therapeutic strategies with oral desmopressin (gradually increased from 0.2 mg three times a day to 0.3 mg four times a day), pituitrin and potassium chloride were of little efficacy, making the diagnosis undetermined. Later, she was speculated to have NDI and was started with extra HCTZ, along with DDAVP gradually reduced to stop. Surprisingly, her polyuria symptom was alleviated and was then given HCTZ twice a day out of the hospital. However, she stopped the treatment on her own and was admitted for polyuria a second time (Table 1).

**Table 1:**
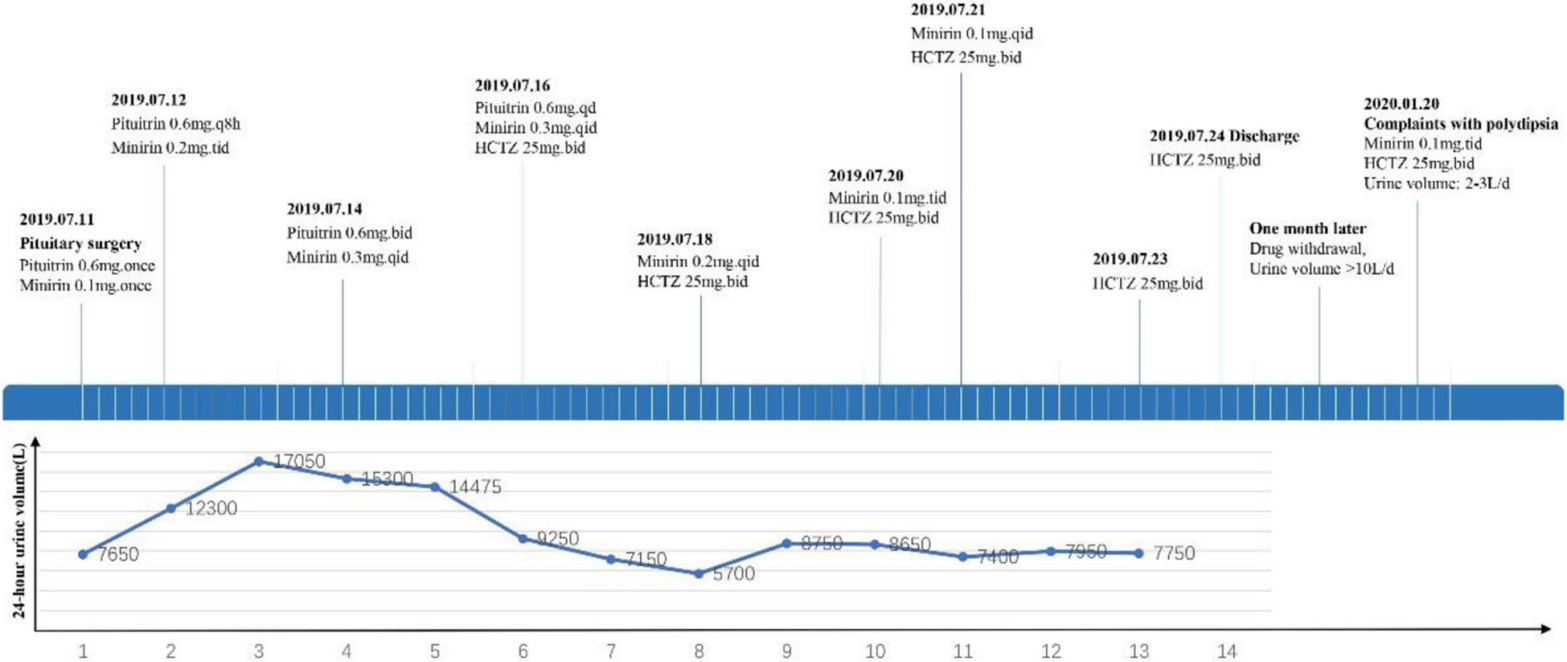
The flowchart of the urine volume and therapeutic strategies in this patient

**Fig. 3:**
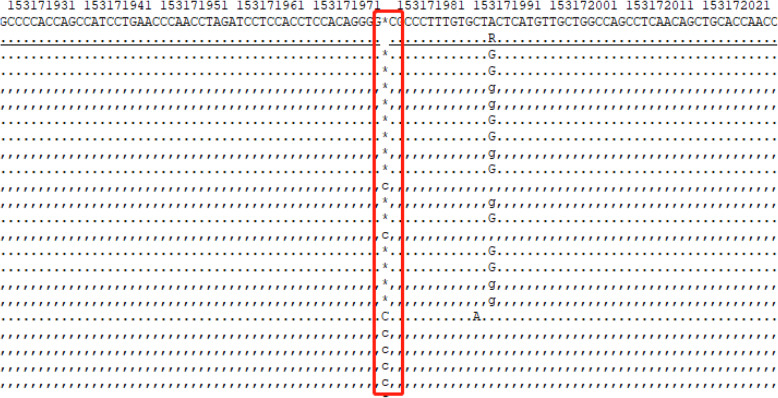
The gene sequencing result of this patient

The physical examination this time revealed that her blood pressure was 108/71 mmHg, with a pulse rate of 121/min. Her skin was dry, and the rest of the physical examination was unremarkable. The laboratory examination revealed the following results: urine specific gravity, 1.002; serum sodium, 134.3 mmol/L; plasma osmolality, and 338.00 mOsm/(kg·H2O). Urinary ultrasonography scans showed normal results. A water deprivation test was performed later with an initial urine gravity of 1.006, plasma osmolality of 281 mOsm/L, and blood pressure of 124/72 mmHg. Two hours after AVP injection, the plasma osmolality was 299.00 mOsm/(kg·H2O), with urine gravity unchanged (more details are shown in Table 2). In consideration of NDI, she was given a genetic test, and the results suggested a heterozygous mutation in arginine vasopressin receptor type 2 (AVPR2) genes *c.914dupC (p.Pro306fs)* **(**Fig. 3**)***.* Further investigation revealed that she had slight polydipsia as well as polyuria for over 20 years. She primarily drank 5 L of water daily along with urine output every 3 h, totaling 4 L per day; however, she did not attach importance to it at that time. As a result of unfavorable therapeutic efficiency with HCTZ during previous discharge, the history of pituitary surgery as well as the slight remission after DDAVP taken during the water depression test, the diagnosis of CNDI and acquired CDI secondary to pituitary surgery should not be excluded, thereby, DDAVP was primarily utilized for 3 days alone for diagnosis therapy, and the treatment was subsequently changed to 25 mg HCTZ twice a day and 0.1 mg DDAVP three times a day. Currently, no recurrence of polyuria or polydipsia has been detected for more than 6 months, indicating the effectiveness of our therapeutic strategies.

**Table 2:** Results of the water deprivation test in this patient

Test time (hour)	Urine volume (ml)	Urine gravity	Weight (kg)	Blood pressure (mm/Hg)	Plasma Osmolality (mOsm/L)
0	400	1.006	62	124/72	281mosm/L
0–0.5	200	1.005	61.5	121/72	NA
0.5–1.5	500	1.004	61	121/73	NA
1.5–2.5	500	1.004	61	116/61	NA
^a^2.5–3	150	1.005	61	106/72	293mosm/L
3–3.5	100	1.005	59	126/73	NA
3.5–4.5	300	1.006	59	112/77	299mosm/L

^a^5 U Vasopressin was administrated at 2.5 h

## Discussion and conclusions

**Fig. 4:**
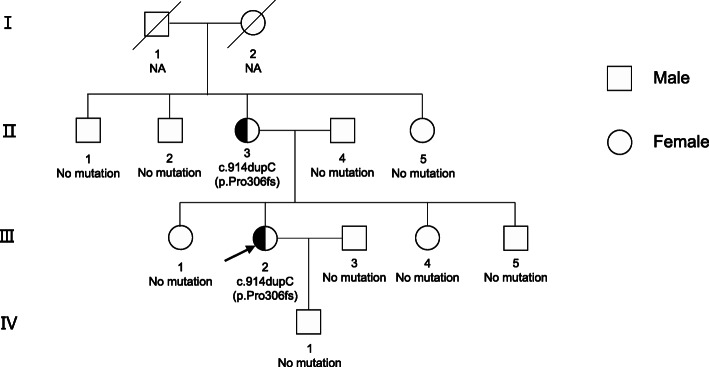
Family trees of patients with congenital nephrogenic diabetes insipidus due to the *AVPR2* mutations

The polyuria and polydipsia symptoms of this patient can be mainly attributed to NDI. Although CDI is likely with damage to vasopressin neurons by pituitary apoplexy or surgery, those patients normally develop polyuria 1–4 days after pituitary surgery and resolve spontaneously; less often, DI is permanent. Therefore, temporary treatment with desmopressin in those patients would be valid [2]. Furthermore, the water deprivation test is the standard way to differentiate polyuria-polydipsia-related diseases. Upon thirst, NDI should be taken into account when urinary osmolality remains < 300 mOsm/kg and does not increase by > 50% after desmopressin injection. On the other hand, complete CDI should be considered if the urinary osmolality increases above 50% after desmopressin injection, whereas in partial CDI, urinary concentration increases to 300–800 mOsm/kg, with an increase in urinary osmolality> 9% [3]. Hereby, our patient presented with sustained urinary osmolality of approximately 300 mOsm/kg, which was mainly indicated by the NDI. In addition, the genetic tests of our patient revealed a heterozygous mutation in AVPR2*_ex3 c.914dupC* (*p.Pro306fs*), which makes the diagnosis of CNDI reliable. It is noteworthy that her mother as well as her grandmother also suffered from polydipsia and polyuria with a low specific urine gravity, despite two brothers and a sister of her mother as well as her grandfather not admitting to polyuria. In addition, this patient had a brother and two sisters who had no polyuria symptoms **(**Fig. 4**)**.

DI is a kind of polyuria-polydipsia syndrome that is characterized by the excretion of large amounts of hypotonic urine. After exclusion of osmotic diuresis, such as diabetes mellitus, DI is normally classified into CDI, NDI and psychogenic polydipsia (PPD) [4]. As a result of the changes in soma volume, hypothalamic neuron-induced vasopressin (VP) is the crucial regulator of water permeability in the collecting ducts [5]. CDI is defined as the impaired production or secretion of VP from the central nervous system [6], while NDI is attributed to the failure of the kidneys to concentrate urine in response to AVP [7, 8]. PPD is common in chronic mental illness patients [9], who present with excessive fluid intake without any potential medical cause [10]. The water deprivation test is essential for differentiating polyuria-related disease [11]. In addition, with MRI of the sella, CDI is characterized by the absence of a posterior pituitary bright spot in T1-weighted imaging, whereas the bright spot is preserved in NDI and PPD; however, an absent bright spot can also be shown in NDI and is possibly attributed to the decreased intracellular AVP stores in the vasopressinergic neurons [12]. Treatment with acquired NDI should target the potential cause, for example, relief of urinary obstruction [13], and the synthetic AVP V2R agonist VP for CDI [14]. Moreover, fluid restriction has become the main strategy in PPD treatment [15].

Recently, more than 250 AVPR2 gene mutations have been identified in the Human Gene Mutation Database [3], among which nearly 55.83% of mutations in the AVPR2 gene are regarded as missense mutation [16]. AVPR2 mutations usually lead to dysregulated receptors, leading to X-linked NDI and impaired urine concentration ability [16]. Approximately 90% of familial NDI is caused by X-linked mutations in AVPR2 and thereby affects males [12], whereas heterozygous females show various degrees of preference [17, 18]. The genetic result of our patient is AVPR2 gene duplication, which leads to a frameshift mutation in the seventh transmembrane domain of exon 3 next to proline 306. Although most NDI patients are found within the first year, some are recognized later due to mild symptoms, especially those with missense mutations [19]. Polyureic states can induce dilation of the urinary tract that leads to impaired bladder function [20]. Thus, the management of dehydration, such as a low-salt diet, thiazide diuretics, and prostaglandin synthesis inhibitors rather than curing the disease, should be emphasized in CNDI [21]. Generally, female patients with AVPR2 mutations tend to exhibit a milder clinical phenotype of partial DI [22]. Here, we may consider polyuria and polydipsia in this patient as pituitary surgery-derived CDI rather than NDI based on the initial clinical manifestation. However, the water depression test as well as the genetic results confirmed it as CNDI. Therefore, direct DNA sequencing of AVPR2 is an accurate approach in helping differentiate the suspected individuals, especially female carriers who present with subtle clinical symptoms.

Although the diagnosis of CNDI accompanied by CDI is conclusive according to her clinical history, the water depression test and genetic results, there still exist several anomalies in this patient. Since she had CDI, the use of Minirin or pituitrin should partially improve polyuria symptoms; nevertheless, there was no significant decrease in urine output after pituitary surgery with large doses of oral vasopressin. Further detailed review of this patient showed that she experienced obvious anxiety during the preoperative period and was given 60 mg of the antianxiety medicine duloxetine as well as 7.5 mg of mirtazapine once a day beginning at the point close to the HCTZ, which also suggested PPD. Unfortunately, as a result of the lack of strict water restriction during that time, the diagnosis of PPD was unreliable. In addition, after elimination of unfavorable emotion as well as withdrawal of the antidepressants, in which there is no spiritual factor interference, the familial NDI along with the CDI should be taken into primary consideration based on the results.

## Data Availability

Not applicable.

## Abbreviations

DI: Diabetes insipidus
CNDI: Congenital nephrogenic diabetes insipidus
CDI: Central nephrogenic diabetes
AVPR2: Arginine vasopressin receptor type 2
HCTZ: Hydrochlorothiazide
NDI: Nephrogenic diabetes insipidus
DDAVP: Desmopressin
MRI: Magnetic resonance imaging
ACTH: Adrenocorticotropic hormone
PPD: Psychogenic polydipsia
